# The role of ciliary function in airway epithelial defense against *Pseudomonas aeruginosa*

**DOI:** 10.1007/s00430-025-00865-9

**Published:** 2025-12-26

**Authors:** Nina Boeck, Philipp Grubwieser, Rudolf Glueckert, Erika Kvalem Soto, Thomas Sonnweber, Alexander Hoffmann, Richard Hilbe, Stefanie Dichtl, Wilfried Posch, Manfred Nairz, Igor Theurl, Zlatko Trajanoski, Guenter Weiss

**Affiliations:** 1 Biocenter, Institute of Bioinformatics, Medical University of Innsbruck, 6020 Innsbruck, Austria; 2 Department of Internal Medicine II (Infectious Diseases, Immunology, Rheumatology, Pulmonology), Medical University of Innsbruck, Anichstraße 35, 6020 Innsbruck, Austria; 3 Institute of Hygiene and Medical Microbiology, Medical University of Innsbruck, 6020 Innsbruck, Austria; 4 Department for Otorhinolaryngology, Head and Neck Surgery, Medical University of Innsbruck, 6020 Innsbruck, Austria; 5 University Clinic for Ear, Nose and Throat Diseases, Tirol Kliniken, 6020 Innsbruck, Austria; 6 Christian Doppler Laboratory for Iron Metabolism and Anemia Research, Medical University of Innsbruck, Innsbruck, Austria

**Keywords:** *Pseudomonas aeruginosa*, Cilia, Roflumilast, Respiratory infection, Organoids

## Abstract

Respiratory cilia play a crucial role in clearing pathogens from the airway, and understanding how *Pseudomonas aeruginosa* (PA) impairs their function is essential for developing targeted therapies to enhance airway epithelial defense and hamper bacterial invasion. Thus, we investigated the mechanisms by which PA impairs respiratory cilia function and identified pharmacological interventions to restore cilia motility. We used mucociliary differentiated human airway organoids expressing motile cilia on the apical side, to model the initial stage of PA infection. We show that co-culturing of organoids with PA down-regulates the expression of genes associated with cilia formation, structure and function. Electron microscopy confirmed ciliary structural damage and membrane disruption in infected organoids and slowed ciliary beating frequency as quantified by slow-motion video recording. Reduction of cilia function resulted in increased bacterial cell invasion, and we identified the bacterial toxin Pyocyanin as one causative cilia damaging factor. We could further demonstrate, that the selective phosphodiesterase-4 inhibitor Roflumilast can maintain ciliary function and beating frequency upon infection of organoids with PA, thereby reducing bacterial invasion of epithelia in our model system. Our findings emphasize the critical role of motile cilia in innate host defense against PA infection, implying that treatment strategies aimed at restoring ciliary function can enhance epithelial robustness during bacterial invasion. Our human organoid infection model enables the evaluation of cellular and functional responses of the airway epithelium to respiratory pathogens and to test potential therapeutic strategies aimed at restoring ciliary function and improving infection control.

**Supplementary Information:**

The online version contains supplementary material available at 10.1007/s00430-025-00865-9.

## Introduction

*Pseudomonas aeruginosa* (PA) is a bacterium of concern because of emerging anti-microbial resistance and due to its potential to cause severe infections, specifically in individuals with chronic lung disease or airway dysfunction. The success of PA as a pathogen can be attributed to various features, including an extensive and adaptable set of virulence factors that damage host cells and facilitate bacterial internalization in airway epithelial cells [1]. Pyocyanin, one such secreted virulence factor, has been studied for its oxidative stress inducing tissue damaging and ciliostatic effects [2]. Pyocyanin reduces cilia beating activity in mammalian airways and thus mucosal clearance of bacteria [3]. While research has focused on exploring pharmacological interventions to block bacterial toxin formation [4–6], little attention has been paid to strategies which improve ciliary function, because the importance of cilia physiology for PA epithelial invasion is incompletely understood. The beating of cilia is partly governed by cyclic adenosine monophosphate (cAMP), which is produced by adenylyl cyclase and degraded by phosphodiesterases (PDEs) [7]. Accordingly, PDE inhibitors, such as Roflumilast, could maintain ciliary beating frequency (CBF) and promote mucosal pathogen clearance [2, 8].

As questions persist regarding alterations in ciliary function by bacteria, and its contribution to host control of infections, a deeper understanding of ciliopathies for pathogen invasion is essential. Thus, advanced cell culture models are needed to comprehend how the loss of motile cilia signaling contributes to bacterial invasiveness and establishment of infection, and how pharmacological rescuing and reinstating of cilia function could potentially limit bacterial multiplication and strengthen host defense in the respiratory tract. Seminal work by Boucher and colleagues [9] established that airway epithelium cultures can generate densely ciliated epithelium with functional mucus transport, forming the basis for many later mechanistic studies.

In addition to these well-differentiated planar airway cultures, which have been widely used to study ciliated airway epithelium, mucociliary transport and ciliary beat frequency, recent organoid technologies provide an additional model system with complementary strengths [10, 11]. Organoids enable long-term expansion of primary airway stem cells and the study of infection dynamics within a three-dimensional epithelial architecture, also retaining many aspects of native cellular heterogeneity and structure of lung tissues. One of their major assets is that patient-derived organoids retain the genetic signature of diseased individuals, enabling the exploration of disease mechanisms and the evaluation of potential treatments aimed at restoring ciliary function in a context closely resembling the clinical condition. Compared with air–liquid interface (ALI) cultures, the apical-out organoid infection model allows infections and pharmacological interventions to be performed under fully submerged and homogeneous liquid conditions, enabling precise control of bacterial inocula and drug exposure [12, 13].

In this study, we established an airway organoid infection model with primary human respiratory cells, including fully differentiated motile cilia. Implementing a bacteria-epithelia co-culture, this model facilitates the study of cilia function during infection. Accordingly, this model was used to examine the effects of PA infection on the structure, motility and function of respiratory cilia, as well as the effects of therapeutic interventions on ciliary beating frequency and bacterial invasion capacity.

## Material and methods

Detailed Methods are provided in the online supplement.

### Organoid culture

Human airway organoids used in this study were purchased from the Foundation Hubrecht Organoid Biobank (www.hubrechtorganoidbiobank.org, HUB code HUB-07-A2-051, normal lung) and were cultured following previously described protocols [10, 14]. The cells were derived from the left superior lobe (lower airway) of a 53-year-old female donor with no underlying lung disease.

### Apical-out polarity switch and mucociliary differentiation of healthy airway organoids

The polarity of the organoids was switched as described by Co et al. [15]. After two days of culturing the organoids in suspension, mucociliary differentiation of organoids was initiated as described by Zhou et al. [16] with the following adaptations: Expansion medium was changed to PneumaCult™-ALI medium supplemented with 2 IU/mL Heparin, 1 µM Hydrocortisone and 10 µM DAPT (Sigma, Cat#D5942). The organoids were then cultured in suspension in a differentiation medium for 16 days.

### Bacterial strains and growth conditions

All experiments were conducted with the *Pseudomonas aeruginosa* PA14 strain, which was generously provided by Dr. Dirk Bumann, Biozentrum Basel, Switzerland. Bacterial cultures were grown as previously published [17].

For experiments with heat-inactivated PA14, bacteria were incubated at 70 °C for 20 min. To confirm the absence of viable bacteria, an aliquot of heat-inactivated PA was plated onto LB plates. In experiments with bacterial cell-free culture supernatant, an overnight culture of PA was centrifuged at 2.000 rcf for 10 min at 4 °C and supernatant was harvested, sterile filtered (0.2 µM), snap-frozen and stored at -80 °C until further usage.

### Treatment with pharmacological compounds

Where indicated, organoids were stimulated with either 10 µM Forskolin (Tocris, Cat#1099/10), 1 µM Roflumilast (MedChem Express, Cat#HY-100639) or 50 µM Pyocyanin (Sigma, Cat# R9532-500UL). In a separate experiment, organoids were exposed to 1 mM EHNA (Tocris, Cat#1261).

### Colony forming unit (CFU) assay

The quantification of viable intracellular bacteria was performed as described before [17]. Total protein was measured using the Micro BCA™ Protein Assay Kit (Thermo Scientific, Cat#23235) according to the manufacturer’s protocol.

### Infection of organoids with PA14 and Gentamicin protection assay

Organoids were infected with 25 × 10^6 bacterial cells/mL Following three hours of active infection, a gentamicin-protection assay was applied to prevent bacterial overgrowth in the cell culture medium. Gentamicin treatment involved washing organoid cultures three times with PBS containing 25 µg/mL gentamicin (Life Technologies, Cat#15710064). For experiments involving gene expression analysis, organoids were incubated in fresh PneumaCult™-ALI medium supplemented with 8 µg/mL gentamicin to effectively eliminate extracellular bacteria, enabling continued culturing of organoids harboring intracellular bacteria [18]. A parallel set of uninfected controls underwent identical washing and incubation procedures. When cells were treated with inactivated bacteria, a concentration of 10^8/mL was used to account for the lack of bacterial growth compared to the viable bacteria during the three-hour active infection phase. Where indicated, organoids were treated with 1:50 (vol/vol) of bacterial cell-free culture supernatant during infection.

### Ciliary beating frequency (CBF) measurements and calculation

Beating cilia were recorded at room temperature (20 °C ± 2 °C) through the ocular lens of an inverted light microscope using 10 × magnification and a camera with high optical and time resolution (1920 × 1080pixels at 240fps, Apple, California) positioned on the eyepiece of the microscope with an adaptor as first described by Chen et al. [19]. One-second movies were collected at 240 frames per second (fps) and saved in.mov format. The software Shutter Encoder was used to convert videos to uncompressed YUV format and videos were imported in the Fiji ImageJ package using the ffmpeg plugin [20]. Kymographs of ciliary movement were created for each video by using the line tool as first described elsewhere [21].

Ten kymographs were randomly selected from each video, depicting the ciliary movement of distinct ciliated cells. The calculation of ciliary beat frequency (CBF) involved manual measurement of the pixels between successive peaks in each kymograph wave. CBF was determined using the following equation:

$$ \left( {{\text{Hz}}} \right) \, = \, \frac{{{\text{Original movie framerate }}\left( {{\text{fps}}} \right)}}{{\text{number of pixels between wave peaks}}} $$

Two independent observers assessed CBF and the measuring method was validated by comparing the results to frequencies acquired using two other techniques. First, with equipment for routine PCD (primary ciliary dyskinesia) diagnosis at the ORL Department. High-speed imaging was performed at 130-300fps depending on the region of interest clipping at 37 °C. Kymographs were extracted with ZEISS ZEN2.6 and CBF was determined as described above. Second, photometric CBF measurement based on the deflection of light caused by the ciliary beats was assessed at 37 °C. The diameter of the photosensitive field on the sample was 5 μm and the light changes photograph-multiplied, digitalized at a sample frequency of 400 Hz, and transformed into a time–amplitude signal. A Fast Fourier transformation analysis was performed every 1.6 s (windaq, Dataq Instruments, Ohio, USA) and CBF values were detected at five to ten different sites. Supplementary Video 1 was recorded at 37 °C for representative visualization of ciliary activity and was not used for CBF measurements.

### mRNA expression analysis of selected genes by RT-qPCR

The RT-qPCR procedure followed established protocols described by Grubwieser et al., 2023 [17]. TaqMan PCR primers were designed with the PrimerQuest™ Tool (Integrated DNA Technologies, Inc., Iowa, USA) and the primer sequences are listed in Supplementary Table S1. The expression levels of individual mRNAs were calculated relative to the housekeeping gene *OAZ1* using the ΔΔCT method.

### Immunofluorescence confocal microscopy of organoid and PA14 co-culture

For immunofluorescence imaging, organoids were infected with fluorescent PA14 and fixed with 4% Paraformaldehyde for 30 min. Next, organoids were permeabilized with 0.5% Saponin (Sigma-Aldrich, Cat#SAE0073) for 30 min, stained with Phalloidin-iFluor 647 (Abcam, Cat#ab176759, 1:1000 dilution) or Alexa Fluor® 647 Anti-alpha Tubulin (acetyl K40) antibody (Abcam, Cat#ab218591, 1:1000 dilution) for 2 h at room temperature (20 °C ± 2 °C) and Fluoroshield Mounting Medium With DAPI (Abcam, Cat#104,139) was added before imaging. Slides were imaged immediately after sample preparation using a VS120-S6 fluorescence microscope (Olympus) or the Operetta CLS System (PerkinElmer, Waltham, MA, USA). Images were captured with a 20-x and 40-x objective using 387/440 nm (DAPI) and 650/684 nm (Alexa-flour-647) lasers and filters, under identical exposure times for every sample.

### Bioinformatic analysis of RNA sequencing data

FASTQ files from 16 samples from 2 different batches were processed with the nf-core RNA-seq pipeline version 3.10.1 [10.5281/zenodo.1400710]. In brief, reads were trimmed with TrimGalore v0.6.10 [10.5281/zenodo.5127899] and were afterwards aligned to the GRCh38 reference genome with GENCODE v38 annotation, using STAR 2.7.10b. Gene expression was quantified using Salmon v1.10.0 [22]. Thereafter, the gene count table was imported into R (v.4.2.3) for downstream analysis. Differential gene expression was performed between infected and control conditions using Bioconductor packages DESeq2 v1.38.3 [23]. To control for batch effect the biological replicate information was added to the DESeq2 design matrix. False-discovery-rates (FDR) were calculated using IHW v1.26 [24]. The filtering of the gene list was performed using threshold values: adjusted P adj.-value < 0.1 and |log2FC|≥ 0.5). The result of the differential expression analysis was used as an input for Gene Set Enrichment Analysis (GSEA) and the over-representation test (ORA). GSEA was performed with the clusterProfiler package v.4.6.2 using the biological processes of the Gene Ontology (GO-BP) database and the Kyoto Encyclopedia of Genes and Genomes (KEGG) as input gene lists. Volcano plots were visualized using the R package EnhancedVolcano v1.16.0. Heatmap plots were generated using ComplexHeatmap v2.14.0. The z-score was calculated by subtracting the mean from the data points and dividing the result by the standard deviation.

### RNA extraction and bulk RNA-sequencing

Organoids were centrifuged at 300 g for 10 min at 4 °C and washed twice on ice with cold PBS. Cell pellets were snap-frozen and stored at − 80 °C until further usage. RNA from approximately 1 × 10^6^ cells was isolated using the RNeasy Plus Micro Kit (Qiagen, Cat#74,034) and the QIAshredder (Qiagen, Cat#79,656) according to the manufacturer’s protocol. Genomic DNA was removed with the RNase-free DNase Set (Qiagen, Cat# 79,254) as per manufacturer’s instructions. RNA was eluted in 1X low Tris–EDTA (TE) buffer, containing 10 mM Tris–HCl and 0.1 mM EDTA. The amount and purity of RNA were measured with a Nanodrop Spectrophotometer. In case of poor quality samples were cleaned using the RNeasy MinElute Cleanup Kit (Qiagen, Cat#74,204) according to the manufacturer’s protocol. RNA Integrity number (RIN) was assessed with the Agilent 2100 Bioanalyzer (Agilent Technologies, Santa Clara, CA, USA). RNA with a RIN > 7.5 was submitted to QuantSeq 3′-mRNA library preparation (Lexogen, Vienna, Austria) and sequenced with an Ion Proton Sequencer using Ion Proton Hi-Q chemistry (Ion Torrent/Fisher Scientific, Austria). Sequencing data obtained from this experiment are also being used in another manuscript 10.3389/fimmu.2024.1508727, https://zenodo.org/records/13382769) and have been deposited on Zenodo 10.5281/zenodo.12648167.

### Electron microscopy

Non-infected and infected organoids were transferred to Eppendorf® vials, passively sedimented and culture media exchanged with Karnovsky’s Glutarladehyde-Formaldehyde fixative. After fixation in 1% aqueous osmium tetroxide and ethanol exposure at ascending concentrations, organoids were embedded in Epon epoxy resin. Additional detail on the method is described elsewhere [25].

### Statistical analysis

Statistical analyses of CBF, CFU and qPCR data were performed using GraphPad Prism v.10.1.2 (San Diego, CA, USA). For comparisons of two groups, unpaired Student’s t-test was used. Comparison of more than two groups was performed using one-way ANOVA followed by Tukey’s post-hoc test for multiple comparisons. Gene expression (multiple groups, multiple time points) was analyzed using two-way ANOVA followed by Sidak’s multiple comparisons test. Differences between groups were considered statistically significant when *p* < 0.05. Correlation between CFU and CBF of infected organoids treated with Forskolin or Roflumilast were calculated using Spearman’s rank correlation test with a 95% confidence interval. The RNA sequencing data was analyzed using R version 4.3.2 as detailed in the supplementary methods.

## Results

### *P. aeruginosa* negatively impacts airway cilia function

To understand the effects of bacterial infection on the function of the respiratory epithelium, we established a human airway organoid infection model with fully differentiated pseudostratified cilia lining the apical side of the epithelium (Supplementary Figure S1a), displaying a synchronized beating movement in a wave-like motion (Supplementary video S1).

**Fig. 1:**
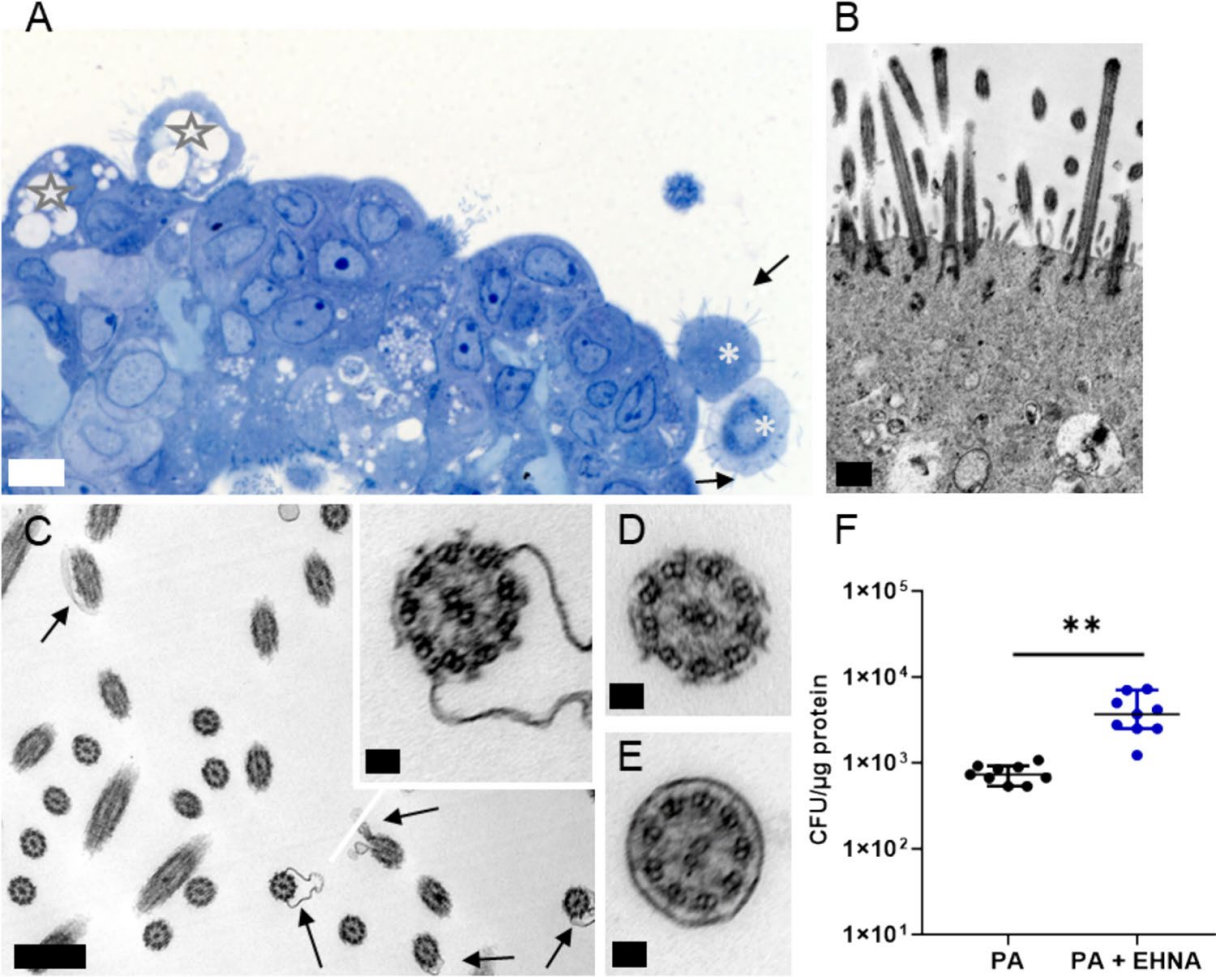
Airway organoids infection with *P. aeruginosa* exhibit ciliary ultrastructure damage, membrane disruption and loss of cilia. **A** Infected organoid showing few ciliated cells and osmotic imbalance (asterisks) and vacuolization (stars). scale bar = 10 µM. **B** shortened cilia. **C** and **D** heavy membrane defects (arrows), **E** compared to axonemes of uninfected organoids. scale bar B,C = 500 nm, D,E,inset C = 50 nm; **F** Quantification of CFUs 3 hpi from organoids infected with PA and infected organoids treated with 1 mM EHNA 2 h before bacterial challenge. Data from three independent experiments are shown as scatter plot with mean ± SD. Paired t-test ** = *p* < 0.002; hpi, hours post infection; PA, *Pseudomonas aeruginosa* strain PA14; EHNA, Erythro-9-(2-hydroxy-3-nonyl)adenine

First, to investigate the effects of bacterial exposure on ciliary function, we added PA directly to the suspension culture and allowed three hours of active infection phase. We then performed electron microscopy to capture the initial impact of direct bacterial contact with cilia. This approach allowed us to observe potential structural damages before the bacteria invaded the epithelium and were subsequently restricted to intracellular compartments as a consequence of gentamicin treatment. We found that exposure to PA caused infection-associated damages in the structure of cilia as evidenced by osmotic disruption of peripheral ciliated epithelia, vacuolization (Fig. 1A) and with some ciliated cells showing fragmented cilia while others maintained their typical appearance (Fig. 1B). Infection also led to a reduction of ciliated cells and ciliary membrane disruption (Fig. 1C and D), compared to organoids that have not been exposed to PA (Fig. 1E). Cilia of uninfected organoids maintained their typical length and their axoneme structure was normal with the typical microtubule arrangement (Supplementary Figure S1b). The observed morphological changes in cilia ultrastructure following bacterial exposure recapitulate observations made in routine diagnostics of patient airway epithelial specimens with bacterial infections. Taken together, these findings reveal that PA causes substantial damage to respiratory cilia. Given this impact, we next sought to determine whether respiratory cilia play a crucial role in limiting bacterial host cell invasion in our organoid infection model. Thus, we quantified viable intracellular bacteria following co-culture with organoids and organoids pretreated with EHNA (erythro-9-(2-hydroxy-3-nonyl)adenine), a dynein inhibitor which impedes cilia beating [26].

PA invaded the organoids and resided intracellularly, whereas organoids pretreated with EHNA prior to bacterial exposure exhibited significantly higher intracellular bacterial numbers compared to untreated PA-infected organoids (Fig. 1F). These results highlight the crucial role of respiratory cilia in innate host defense against bacterial epithelial invasion, and suggest that cilia function is a decisive component of the host–pathogen-interplay and that its disruption is a possible target for invading PA.

### *P. aeruginosa* infection impairs transcriptional regulation of ciliogenesis and cilia motility in airway epithelial cells

**Fig. 2:**
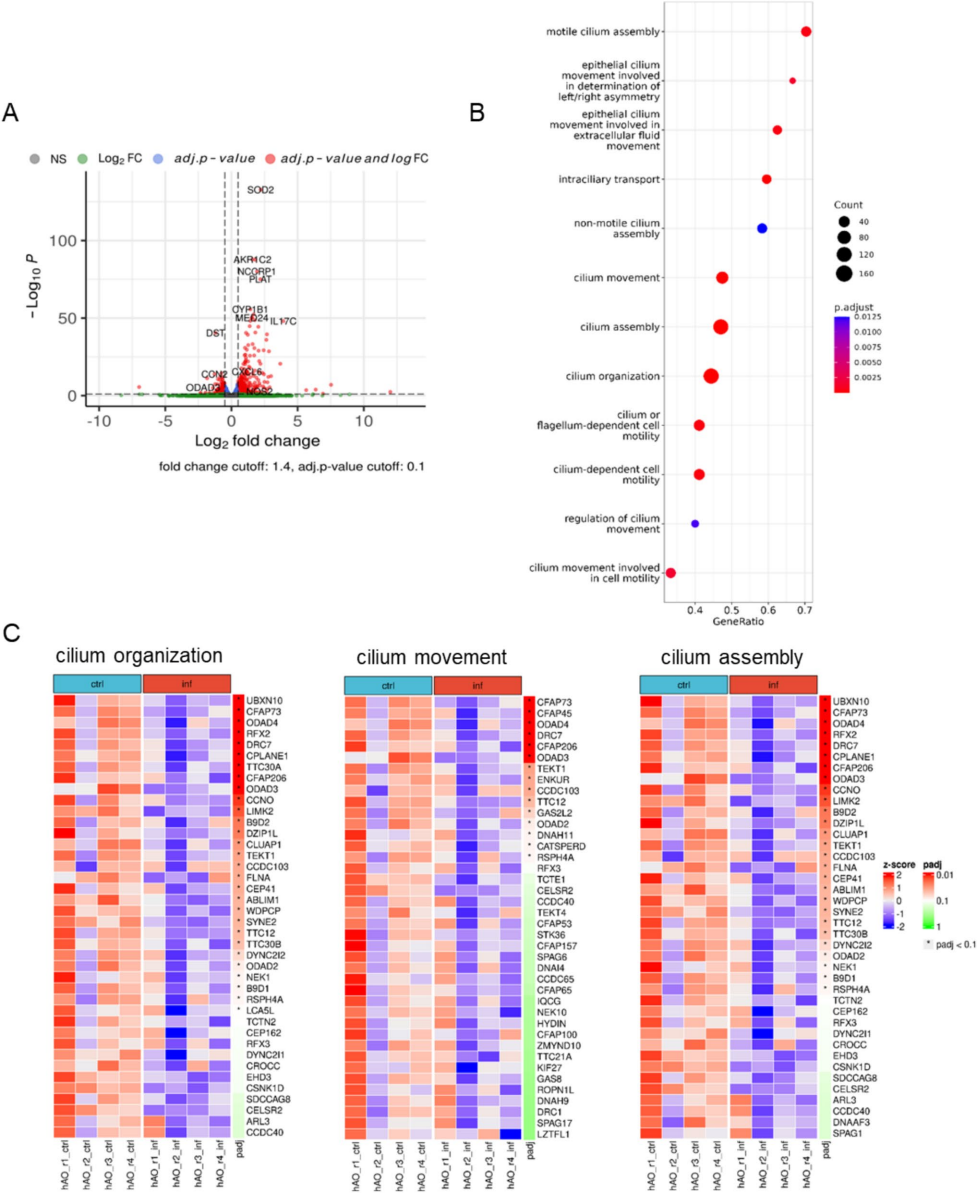
Infected organoids show significant downregulation of genes attributed to cilia assembly and ciliary motility. **A** Volcano plot showing the log_2_ fold change and -log_10_ adjusted p-value on the x-axis and y-axis respectively of the differentially expressed genes (525 genes) of PA-infected versus uninfected organoids 7 hpi. **B** Gene Set Enrichment Analysis of organoids exposed to PA compared to uninfected organoids showing top 12 gene sets derived from the Gene Ontology database Biological Process. Color coding by adjusted p-values **C** Heatmaps depicting altered gene expression of gene sets involved in cilium organization (GO term category GO:0044782, left panel), cilium movement (GO term category GO:0003341, middle panel) and cilium assembly (GO term category GO:0060271, right panel) in PA-14 infected versus uninfected organoids 7 hpi. The z-score and adjusted p-value are color-coded. hpi, hours post infection

To further elucidate the effect of PA infection on ciliary function, we compared the transcriptional profile of PA-infected organoids to that of uninfected organoids in order to identify infection-related changes in gene expression at four hours after gentamicin-protected infection (= 7 h post infection, hpi). To do so, we applied a gentamicin treatment after the active infection phase to remove extracellular bacteria. This approach was chosen because PA is known to invade the epithelium, thus it is crucial to model both the extracellular and intracellular phases of infection to capture the entire infection dynamics and understand the pathogen’s impact on airway epithelial cell responses depending on the cellular localization of the bacterium [27]. Principal-component analysis (PCA) demonstrated close similarities among samples belonging to the same treatment group after correction for batch effects (Supplementary Figure S2a). Infected airway organoids exhibited an extensively altered transcriptional activation profile compared to uninfected controls (Fig. 2A), with 525 genes differentially expressed (DEGs; 343 upregulated, 182 downregulated, Supplementary Table S1). Gene Set Enrichment Analysis of PA-infected organoids demonstrated significantly downregulated gene sets associated with cilia formation, structure and function when compared to non-infected organoids (Fig. 2B). Of special interest, infection resulted in the downregulation of a substantial number of genes involved in cilium organization, movement and assembly, suggesting a causative role of PA for ciliary dysfunction (Fig. 2C). The differential expression of selected ciliary genes *ODAD3*, *EHD3*, *CELSR2* and *KIF24* was additionally confirmed by RT-qPCR. To further investigate the temporal dynamics of these transcriptional changes, we also analyzed later time points of infection (8 h and 16 h). Those results also revealed a downregulation of *CELSR2*, EHD3, *KIF24* and *ODAD3*, indicating that the observed inhibition in regulation of these genes is sustained over time and not merely a transient response, thus potentially contributing to long-term ciliary dysfunction (Supplementary Figure S2b). Taken together, these findings highlight the direct and lasting impact of PA infection on host gene expression patterns involved in cilia biology, particularly cilia motility, suggesting that dysfunction of respiratory cilia may be an early hallmark during bacterial invasion and a potential pathogen-induced mechanism that may promote bacterial access to the epithelial surface.

### Ciliary beating frequency is drastically reduced in infected organoids and impaired cilia motility is mediated by the secreted PA virulence factor Pyocyanin

**Fig. 3:**
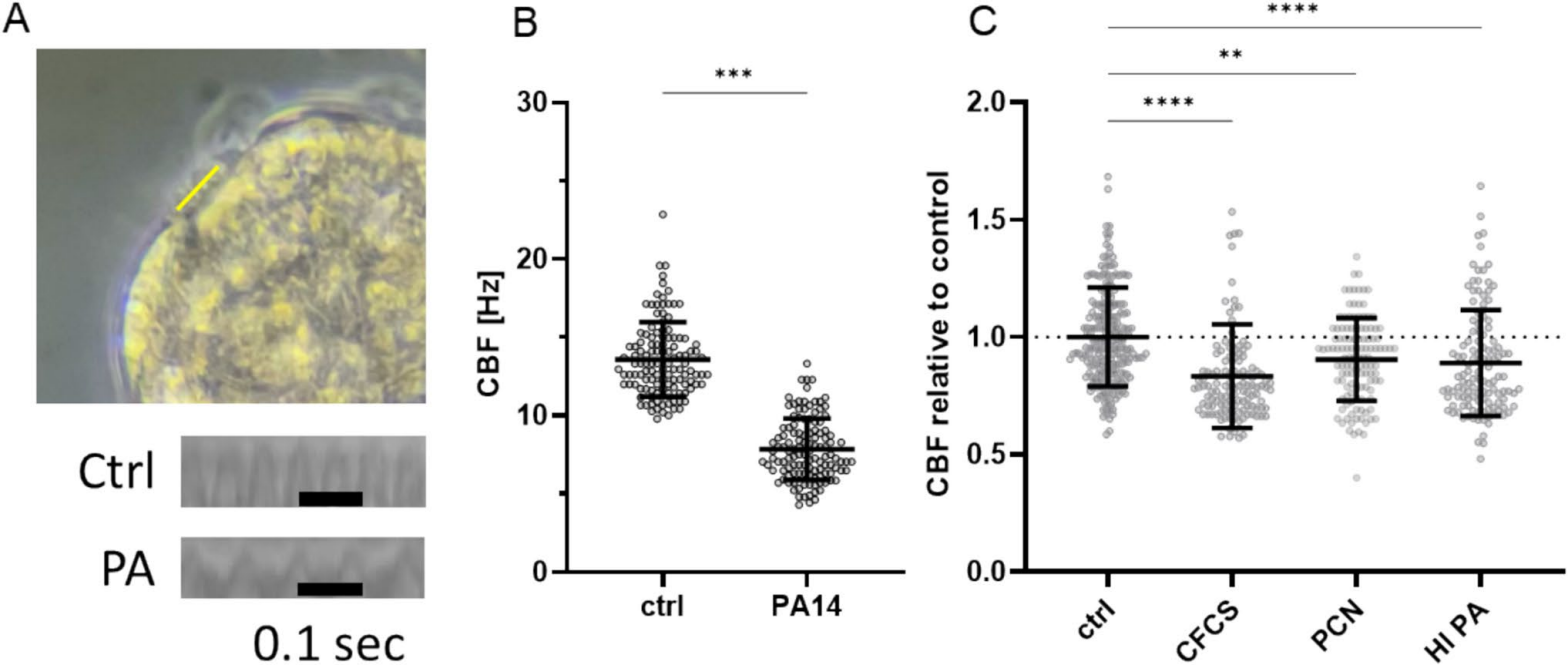
Co-culture with *P. aeruginosa* and bacterial-derived compounds causes respiratory cilia dysfunction by reducing ciliary beating frequency in organoids. **A** A frame of an exemplary video recording of organoids and the selected line (yellow) drawn through the beating cilia for Kymograph image generation are shown in the top panel. Representative Kymographs are shown below for control- as well as PA exposed organoids. Black bar represents 0.1 s. fps, frames per second. **B** Quantification of ciliary beat frequency (CBF) in Hertz (Hz) of uninfected and PA-infected organoids. Data are shown as individual values ± SD. Minimum 120 observations from 3 independent experiments. Two-tailed unpaired t-test. *** = *p* < 0.0001. **C** CBF measurements of organoids treated with PA-derived cilia-inhibiting compounds normalized to uninfected controls. Data are shown as scatter plot with individual values ± SD with dotted line at y = 1. Minimum 120 observations from 3 independent experiments. ** = *p* < 0.002, **** = *p* < 0.0001 for one-way ANOVA with post-hoc statistical testing. PA, *Pseudomonas aeruginosa* strain PA14; CFCS = bacterial cell free culture supernatant, PCN = Pyocyanin, HI PA = heat inactivated PA

Since the transcriptomic analysis revealed extensive changes in gene expression associated with cilia structure and function following organoid-bacteria co-culture, we next assessed the impact of PA infection on cilia motility three hours post infection by quantifying CBF with slow-motion video recordings. Therefore, beating cilia were recorded at a sampling rate of 240 frames per second (fps) and CBF was calculated from kymographs as described before (Fig. 3A) [21]. Cilia lining the epithelium of uninfected airway organoids exhibited a CBF of 13.6 ± 2.4 Hz (Fig. 3B), which is in the expected physiological range, highlighting the physiological relevance of our infection model [28, 29]. In contrast, cilia of infected organoids exhibited significantly reduced CBF (7.8 ± 2.0 Hz) when compared to cilia of organoids that were not exposed to PA (Fig. 3B). Notably, the results of the video analysis of cilia beating frequency were confirmed by two additional standard quantification techniques for cilia motility (Supplementary Fig. 3). To narrow down which PA-derived compounds are responsible for CBF reduction, we next tested whether soluble factors secreted by the pathogen or surface components of the outer bacterial membrane are inducing the observed drop in CBF. By incubating organoids with bacterial cell-free culture supernatant, the PA-derived toxin Pyocyanin or heat-inactivated PA we observed that all those treatments lead to a significant decrease in CBF compared to mock-treated organoids (Fig. 3C). These findings suggest that both, soluble factors secreted by PA and bacterial cell wall components are capable of inducing motile cilia functional impairment in lung organoids and that the PA-derived toxin Pyocyanin is one causative virulence factor responsible for this effect.

### Functional impairment of respiratory cilia due to *P. aeruginosa* infection is restored by adenylyl cyclase activation and phosphodiesterase-4 inhibition

**Fig. 4:**
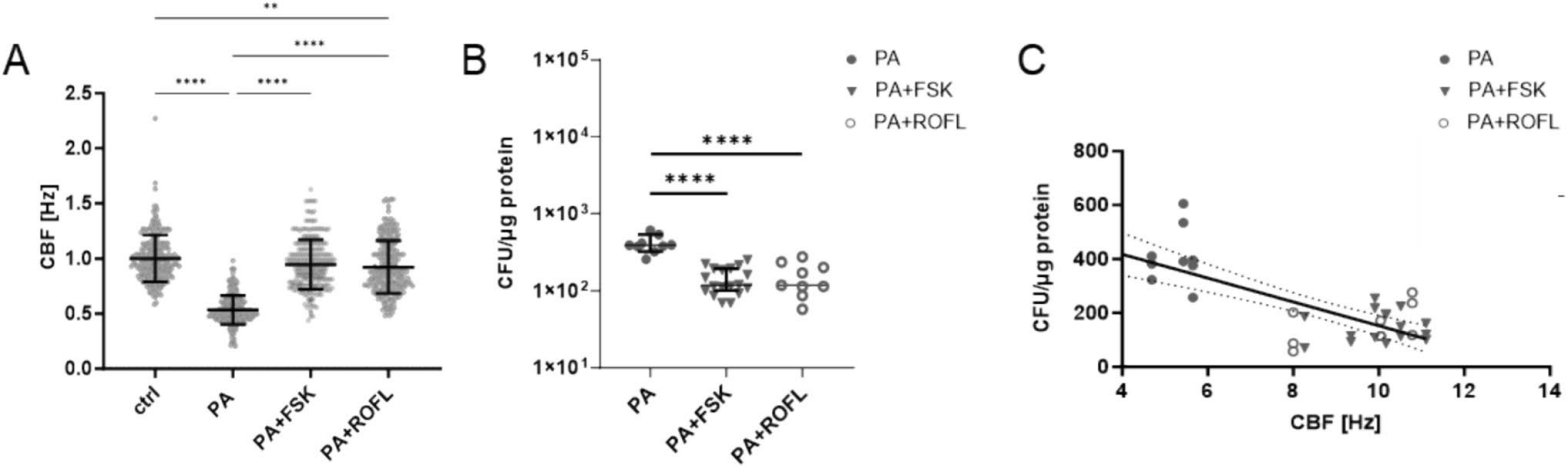
Drug treatment of organoids prior to infection boosts cilia motility and decreases bacterial load. **A** Mean CBF values of uninfected organoids, PA-14 infected organoids and organoids pretreated with 10 µM Forskolin or 1 µM Roflumilast prior to infection **B** Quantification of CFU following infection of organoids with PA and infected organoids treated with 1 µM FSK or 1 µM ROFL during organoid-bacteria co-culture **C** Spearman’s rank correlation depicting the association between CFU shown on the y-axis and CBF on the y-axis. Data measured 3 hpi are shown as individual results and linear regression line (r = 0.51; *p* < 0.0001). hpi, hours post infection; PA, *Pseudomonas aeruginosa* strain PA14; FSK, Forskolin; ROFL, Roflumilast

Next, we aimed to resolve cilia dysfunction following PA infection by testing whether modulating cAMP levels could improve the observed reduction in CBF, since cAMP plays a crucial role in regulating ciliary activity [7]. Thus, we incubated organoids with Forskolin, which increases intracellular cAMP levels by stimulating its production through activating adenylyl cyclase [30] and Roflumilast, which helps to maintain cAMP level by preventing its degradation by PDE [31] and quantified CBF 3 hpi after pharmacological intervention. Treatment of lung organoids with Forskolin resulted in elevated CBF values when compared to mock-treated infected organoids (10.2 ± 2.5 Hz vs. 5.1 ± 1.1 Hz; *p* < 0.0001). Similarly, Roflumilast increased the CBF of treated organoids significantly compared to untreated infected organoids (9.2 ± 2.2 Hz vs. 5.1 ± 1.1 Hz; *p* < 0.0001) (Fig. 4A). To investigate whether restored ciliary function prevents bacterial entry, we next quantified the number of viable intracellular bacteria. Interestingly, we observed a significant decrease in intracellular CFU counts when organoids were incubated with Forskolin or Roflumilast (Fig. 4B). An inverse correlation was found between CFU and CBF values, with higher CFU counts in untreated infected organoids negatively correlating with lower CBF values and conversely, lower CFU counts in infected organoids treated with Forskolin and Roflumilast strongly correlating with higher CBF values 3 hpi (*p* < 0.0001, Spearman’s rank r = 0.51) (Fig. 4C). These results indicate that Roflumilast and Forskolin both have a stimulatory effect on cilia functionality, and can reverse PA-induced reduction of cilia motility as quantified by CBF decrease. Moreover, by improving cilia function, Forskolin and Roflumilast were capable of hampering bacterial host cell invasion resulting in lower intracellular bacterial numbers. While these data demonstrate a clear inverse relationship between ciliary motility and epithelial invasion in our organoid infection model, this does not provide a definitive proof of a cause-effect relationship between these two observations, and requires further elucidation regarding its physiological relevance.

## Discussion

In this study, we demonstrate how PA infection disrupts ciliary structure and function in a human airway organoid infection model featuring a motile cilia lining. Our findings further show that PA-induced ciliary impairment can be attenuated by pharmacological restoration of CBF, providing a proof-of-concept that ciliary dysfunction is, at least in part, reversible in this system. Given that drugs like Roflumilast, with the capacity to restore ciliary function, are already approved for clinical use, our findings provide a mechanistic basis for exploring whether such approaches could complement existing antimicrobial therapies and help to improve epithelial resilience in individuals with chronic pulmonary diseases who experience recurrent infection episodes or PA colonization.

In line with our findings, impaired ciliary movement was also reported in a study carrying out transcriptomic profiling of PA-infected chronic obstructive pulmonary disease (COPD) nasopharyngeal organoids [32]. Similar to in vitro findings with ciliated human bronchial epithelial (HBE) cells compromised by exposure to cigarette smoke extracts [31, 33], infected organoids pretreated with Roflumilast showed increased CBF in our experiments. A study from the ‘90 s using human nasal epithelial brushings found that the CBF slowing caused by Pyocyanin could be significantly inhibited by compounds like Forskolin, which increase or stabilize intracellular ATP and cAMP levels [3]. In that study, Pyocyanin was shown to decrease intracellular cAMP and ATP, providing a plausible mechanistic basis for its cilia-inhibitory effects. As we did not measure intracellular cAMP in our model, future studies examining cAMP levels in Pyocyanin-treated airway organoids would help to define the precise intracellular mechanism underlying ciliary impairment. Roflumilast, used in clinics to treat COPD patients, may help to maintain CBF and promote pathogen clearance during airway infections, since such PDE inhibitors are known to prevent PDE-mediated degradation of intracellular cAMP [8].

To the best of our knowledge, this study is the first to demonstrate the pharmacological reversal of ciliary impairment following PA infection. Specifically, we show that Roflumilast reverses PA-induced cilia function impairment and combats bacterial invasion of the lung epithelium. To date, there are no research studies that have investigated whether pharmacological intervention can prevent the cilio-inhibiting effects of virulence factors secreted by PA. An in vitro study working with air–liquid interface cultures showed that treatment with Roflumilast prevented respiratory syncytial virus infection in differentiated HBE cells and prevented loss of ciliated cells [34]. Taken together, our results strongly suggest that soluble adenylyl cyclase and phospho-diesterase-4 are druggable targets to enforce epithelial defense in PA infection. While Roflumilast restored ciliary motility and reduced bacterial invasion, it remains to be determined whether this functional rescue is accompanied by preservation of ciliary ultrastructure or mitigation of infection-induced transcriptional changes. Furthermore, it will be important to test whether this is also true in vivo and relevant also for other highly virulent pathogens.

The model presented here may also be highly relevant for studying patients with chronic lung diseases, characterized by impaired ciliogenesis and altered ciliary movement, as these individuals are highly susceptible to respiratory infections with PA. Specifically, our discovery can be of importance for patients who are chronically colonized with PA, such as those with cystic fibrosis, to prevent pathogen invasion and reduce the risk of pulmonary infection. Furthermore, viral and bacterial infections are a major trigger of exacerbations in COPD patients [35], highlighting the urgent need for additional treatment options. Strengthening the immunological and barrier functions of the respiratory epithelium is a promising treatment target. This approach may be particularly useful as an adjunct therapy to anti-infective treatments, and it may avoid bacterial re-colonization or reduce the risk of re-infection. Roflumilast is particularly effective in COPD patients who experience frequent exacerbations and exhibit an inflammatory disease phenotype [36]. While the effectiveness in reducing inflammation can be attributed to its mode of action, our study suggests that its ability to reduce exacerbations may also be due to its positive effects on cilia function and the prevention of bacterial invasion. For instance, airway organoids derived from cystic fibrosis (CF) patients could serve as a valuable tool to study cilio-inhibitory events caused by PA infection and their consequences on bacterial load.

Limitations of this study include the absence of a continuous mucus layer in our infection model, which prevents assessment of mucociliary transport rate (MTR). Changes in CBF cannot be assumed to mirror or predict changes in MTR, since MTR also depends on mucus properties that are not present in apical-out organoids. Consequently, we cannot draw conclusions about mucociliary clearance or mucus-dependent bacterial removal. Notably, cAMP-modulating agents can exert complex and context-dependent effects on MTR, as demonstrated in native tissues, where Forskolin alone did not enhance MTR but acted synergistically with carbachol [37]. Future studies incorporating mucus-producing airway models or in vivo infection models will be required to determine how restored CBF relates to mucociliary transport and bacterial clearance under physiological conditions.

Moreover, because our apical-out organoid system does not generate a continuous mucus layer, the reduction in bacterial invasion observed at higher CBF values reflects the epithelial-intrinsic contribution of motile cilia once bacteria are already in close proximity to the cell surface. In vivo, the mucus layer constitutes the first protective interface and would be expected to further restrict bacterial access to epithelial cells. Our model therefore does not aim to reproduce the full mucociliary clearance apparatus, but instead indicates the direct contribution of ciliary motility to limiting bacterial invasion under conditions where pathogens have already reached the epithelial surface.

Because all airway organoids were derived from one single donor, the study does not capture the biological variation that may exist across individuals. Future work using organoids from multiple independent healthy but also diseased donors will be important to validate the generalizability and robustness of the observed effects.

## Supplementary Information

Below is the link to the electronic supplementary material.

Supplementary file1 (DOCX 918 kb)

Supplementary file2 (PPTX 11424 kb)

## Data Availability

Sequencing data obtained from this experiment are also being used in another manuscript 10.3389/fimmu.2024.1508727, https://zenodo.org/records/13382769) and have been deposited on Zenodo 10.5281/zenodo.12648167.
