# Supplementary material for: The role of ciliary function in airway epithelial defense against Pseudomonas aeruginosa
Source: Med Microbiol Immunol. 2025 Dec 26;215(1):3. doi: 10.1007/s00430-025-00865-9 (PMC12743103; doi:10.1007/s00430-025-00865-9)
Supplement: Supplementary file 1 — Supplementary file1 (DOCX 918 kb) [file 430_2025_865_MOESM1_ESM.docx]

The Role of Ciliary Function in Airway Epithelial Defense against *Pseudomonas aeruginosa*

Nina Boeck^1*^, Philipp Grubwieser^2,3*^, Rudolf Glueckert^4,5^, Erika Kvalem Soto^1^, Thomas Sonnweber^2^, Alexander Hoffmann^2^, Richard Hilbe^2^, Stefanie Dichtl^3^, Wilfried Posch^3^, Manfred Nairz^2^, Igor Theurl^2^, Zlatko Trajanoski^1^, Guenter Weiss^2,6,7^

1 Biocenter, Institute of Bioinformatics, Medical University of Innsbruck, Innsbruck, Austria.

2 Department of Internal Medicine II, Infectious Diseases, Immunology, Rheumatology, Pulmonology, Medical University of Innsbruck, Innsbruck, Austria.

3 Institute of Hygiene and Medical Microbiology, Medical University of Innsbruck, Innsbruck, Austria.

4 Department for Otorhinolaryngology, Head and Neck Surgery, Medical University of Innsbruck, Innsbruck, Austria.

5 University Clinic for Ear, Nose and Throat Diseases, Tirol Kliniken, 6020 Innsbruck, Austria

6 Christian Doppler Laboratory for Iron Metabolism and Anemia Research, Medical University of Innsbruck, Innsbruck, Austria.

* These authors contributed equally

7 Corresponding Author

_Correspondence should be addressed to Guenter Weiss, M.D., Prof. Department of Internal Medicine II, Infectious Diseases, Immunology, Rheumatology, Pulmonology, Medical University of Innsbruck, Anichstraße 35, 6020 Innsbruck, Austria. E-mail: guenter.weiss@i-med.ac.at_

Supplementary Table S1: TaqMan primers used in this study (sequences from 5′ to 3’):

| target | forward primer | reverse primer |
| --- | --- | --- |
| *EHD3* | ATTACCGCTTCCACGAGTTC | CAATCCTCATGCCTGGGAAG |
| *KIF24* | GTCTACTGGCTCTGAAGGAAT | TGGCTTGGTGAGATGTTGG |
| *ODAD3* | AAGAAACTGGAGAACGAGCG | CCAAAGATCACCTCCATCTGG |
| *CELSR2* | TCTGGAACCATTCAATCCTGG | AGAAACGTCCATGAGCACAG |
| *OAZ1* | GGATCCTCAATAGCCACTGC | TACAGCAGTGGAGGGAGACC |


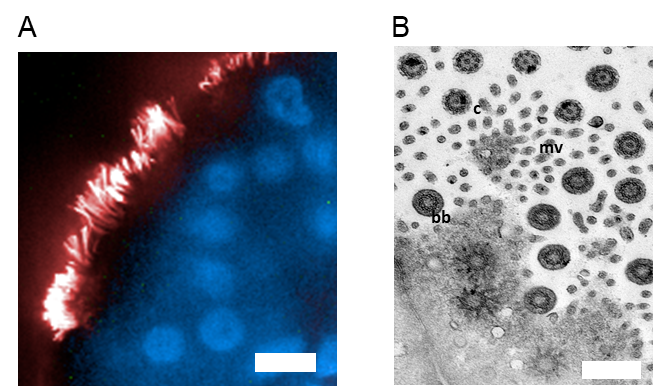


**Supplementary figure S1** Human Airway Organoids with Motile Cilia Mimic Native Respiratory Epithelium to Study the Interplay between Cilia and Pathogens

(A) Cilia expression on the apical side of airway organoids after mucociliary differentiation was confirmed by IF staining with anti-acetylated α-tubulin (Ac-α-Tub, red) antibody. Scale bar = 10 µm (B) Electron microscopy depicting typical cilia morphology in uninfected control organoids with normal basal body (bb) and ciliary axoneme structure surrounded by microvilli (mv). Scale bar = 500 nm


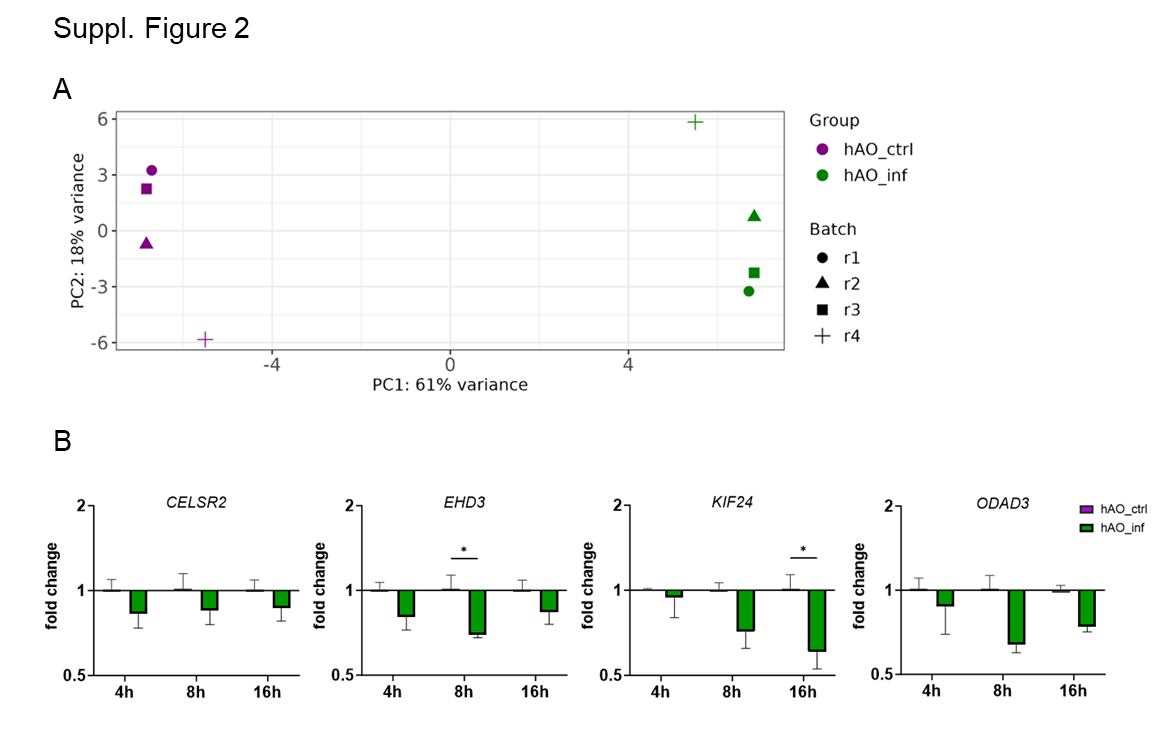
**Supplementary figure S2** Impact of P. aeruginosa infection on host epithelial gene expression (A) Principal Component Analysis after batch effect correction. Point Plot of two principal components which were mapped to treatment. Groups include uninfected organoids (hAO_ctrl, purple) and *Pseudomonas aeruginosa*-infected organoids (hAO_inf, green). Symbols refer to different batches (n=4) (B) mRNA expression levels of selected genes involved in cilia organization in infected organoids normalized to uninfected control organoids. RNA was extracted 4, 8 and 16 hrs after Gentamicin-protected infection.  Data shown as mean ± SD of a triplicate experiment. * = p < 0.05; two-way ANOVA with Sidak’s multiple comparisons test. hAO, human airway organoid; ctrl, uninfected control; inf, infected with PA14 Pseudomonas aeruginosa strain


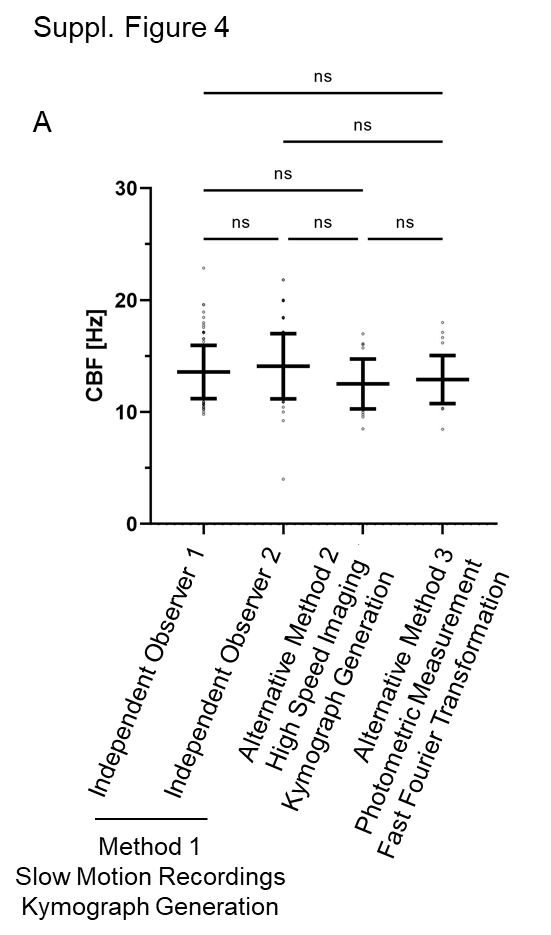


**Supplementary figure S3** Validation of Measurement Method for Quantification of Ciliary Beating Frequency

Ciliated organoid samples were analyzed for Ciliary beating frequency (CBF) by three different methods. For Method 1 slow motion recordings were used to generate Kymographs and assessed by two independent observers. For Alternative Method 2 High Speed Images were recorded and Kymographs were generated. For Alternative Method 3 a photometric signal was processed using a Fourier transform to show the CBF distribution


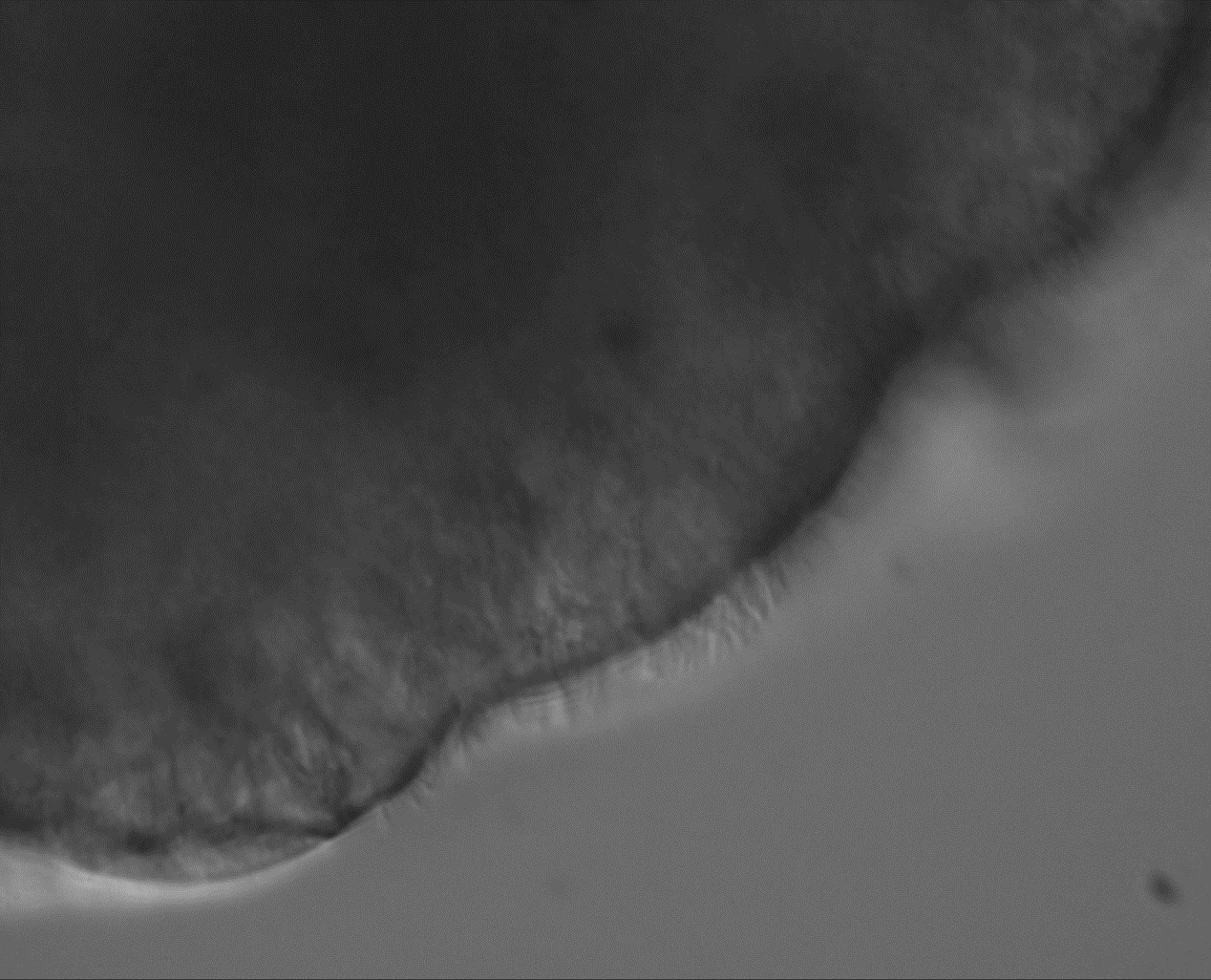


**Supplementary Video 1** Airway Organoids Express Motile Cilia on their Exterior Representative video recording demonstrating the presence of multiciliated cells on the outer side of human airway organoids. The cilia exhibited coordinated, wave-like beating patterns. Video was recorded at 37°C using a Zeiss Axiovert A1 microscope with a 40x objective
