# Supplementary material for: The role of ciliary function in airway epithelial defense against Pseudomonas aeruginosa
Source: Med Microbiol Immunol. 2025 Dec 26;215(1):3. doi: 10.1007/s00430-025-00865-9 (PMC12743103; doi:10.1007/s00430-025-00865-9)
Supplement: Supplementary file 2 — Supplementary file2 (PPTX 11424 kb) [file 430_2025_865_MOESM2_ESM.pptx]

## Slide 1
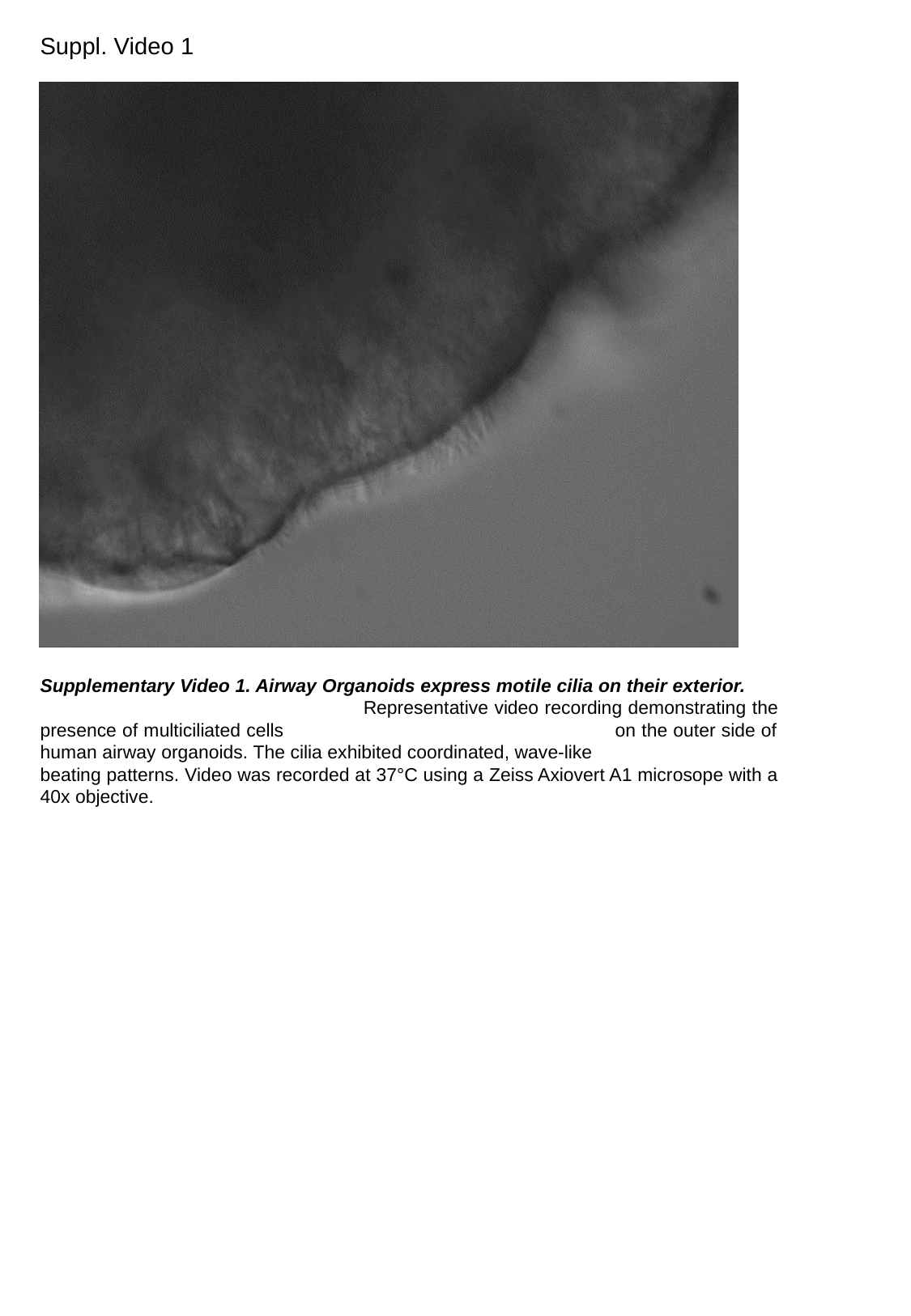

Suppl. Video 1
Supplementary Video 1. Airway Organoids express motile cilia on their exterior. Representative video recording demonstrating the presence of multiciliated cells on the outer side of human airway organoids. The cilia exhibited coordinated, wave-like beating patterns. Video was recorded at 37°C using a Zeiss Axiovert A1 microsope with a 40x objective.
